# Supplementary material for: From sequence to enzyme mechanism using multi-label machine learning
Source: BMC Bioinformatics. 2014 May 19;15:150. doi: 10.1186/1471-2105-15-150 (PMC4229970; doi:10.1186/1471-2105-15-150)
Supplement: Additional file 2 — Java code of ml2db. Additional file ml2db_code.tar.gz contains the Java source code to run the multi-label machine learning experiments and save the results to database. The code’s Javadoc is included. [file 1471-2105-15-150-S2.zip › additional file 2/ml2db/ml2db/doc/main/ml2db/mulan/learn/traintest/TrainTestEvaluator.html]

TrainTestEvaluator


JavaScript is disabled on your browser.


- Overview
- Package
- Class
- Use
- Tree
- Deprecated
- Index
- Help

- Prev Class
- Next Class

- Frames
- No Frames

- All Classes

- Summary:
- Nested |
- Field |
- Constr |
- Method

- Detail:
- Field |
- Constr |
- Method


main.ml2db.mulan.learn.traintest

## Class TrainTestEvaluator

- java.lang.Object
- - mulan.evaluation.Evaluator
  - - main.ml2db.mulan.learn.evaluation.EvaluatorNoAUC
    - - main.ml2db.mulan.learn.crosseval.CrossEvaluator
      - - main.ml2db.mulan.learn.traintest.TrainTestEvaluator

- Direct Known Subclasses:
  :   TrainTestEvaluatorSerialised

  ---

    

  ```
  public class TrainTestEvaluator
  extends CrossEvaluator
  ```

  Train/test evaluator. The learner can be trained/built on the spot or can be
  taken from a file containing an already trained/build serialised learner.

  Version:
  :   21 Oct 2010

  Author:
  :   Luna De Ferrari ldf(at)st-andrews.ac.uk

- - ### Constructor Summary

    Constructors

    Constructor and Description

    `TrainTestEvaluator(TrainTestExperimenter experimenter)`
  - ### Method Summary

    Methods

    | Modifier and Type | Method and Description |
    | --- | --- |
    | `TrainTestExperimenter` | `getExperimenter()` |
    | `PredictionsSaver` | `getPredSaver()` |
    | `java.util.Vector<java.lang.Object[]>` | `trainTestValidate(IdMultiLabelInstances trainSet, IdMultiLabelInstances testSet, boolean savePredictions, boolean saveTrueNegatives)` |

    - ### Methods inherited from class main.ml2db.mulan.learn.crosseval.CrossEvaluator

      `evaluate, internallyCrossValidate`
    - ### Methods inherited from class main.ml2db.mulan.learn.evaluation.EvaluatorNoAUC

      `evaluate, evaluate, getEndTime, getStartTime, getStrictness, isStrict, setEndTime`
    - ### Methods inherited from class mulan.evaluation.Evaluator

      `checkData, checkLearner, crossValidate, crossValidate, evaluate, evaluate, setSeed`
    - ### Methods inherited from class java.lang.Object

      `equals, getClass, hashCode, notify, notifyAll, toString, wait, wait, wait`

- - ### Constructor Detail


    - #### TrainTestEvaluator

      ```
      public TrainTestEvaluator(TrainTestExperimenter experimenter)
      ```
  - ### Method Detail


    - #### getExperimenter

      ```
      public TrainTestExperimenter getExperimenter()
      ```

      **Overrides:**
      :   `getExperimenter` in class `EvaluatorNoAUC`


    - #### getPredSaver

      ```
      public PredictionsSaver getPredSaver()
      ```


    - #### trainTestValidate

      ```
      public java.util.Vector<java.lang.Object[]> trainTestValidate(IdMultiLabelInstances trainSet,
                                                           IdMultiLabelInstances testSet,
                                                           boolean savePredictions,
                                                           boolean saveTrueNegatives)
      ```

      Parameters:
      :   `trainSet` - the training set
      :   `testSet` - the test set
      :   `savePredictions` - whether to emit predictions and save them to database

      Returns:
      :   an array containing: run number (String), startOfRun (sql data as
          String), endOfRun (sql date as String), algorithm, train set id,
          test set id, evaluation results (mulan.Evaluation)


- Overview
- Package
- Class
- Use
- Tree
- Deprecated
- Index
- Help

- Prev Class
- Next Class

- Frames
- No Frames

- All Classes

- Summary:
- Nested |
- Field |
- Constr |
- Method

- Detail:
- Field |
- Constr |
- Method
